# piRBase: a web resource assisting piRNA functional study

**DOI:** 10.1093/database/bau110

**Published:** 2014-11-23

**Authors:** Peng Zhang, Xiaohui Si, Geir Skogerbø, Jiajia Wang, Dongya Cui, Yongxing Li, Xubin Sun, Li Liu, Baofa Sun, Runsheng Chen, Shunmin He, Da-Wei Huang

**Affiliations:** ^1^Key Laboratory of the Zoological Systematics and Evolution, Institute of Zoology, Chinese Academy of Sciences, Beijing 100101, China, ^2^University of Chinese Academy of Science, Beijing 100049, China, ^3^Laboratory of Bioinformatics and Noncoding RNA, Institute of Biophysics, Chinese Academy of Sciences, Beijing 100101, China, ^4^College of Life Sciences, Hebei University, Baoding 071002, Hebei, China and ^5^College of Plant Protection, Shandong Agricultural University, Tai'an 271018, Shandong, China

## Abstract

piRNAs are a class of small RNAs that is most abundantly expressed in the animal germ line. Presently, substantial research is going on to reveal the functions of piRNAs in the epigenetic and post-transcriptional regulation of transposons and genes. A piRNA database for collection, annotation and structuring of these data will be a valuable contribution to the field, and we have therefore developed the piRBase platform which integrates various piRNA-related high-throughput data. piRBase has the largest collection of piRNAs among existing databases, and contains at present 77 million piRNA sequences from nine organisms. Repeat-derived and gene-derived piRNAs, which possibly participate in the regulation of the corresponding elements, have been given particular attention. Furthermore, epigenetic data and reported piRNA targets were also collected. To our knowledge, this is the first piRNA database that systematically integrates epigenetic and post-transcriptional regulation data to support piRNA functional analysis. We believe that piRBase will contribute to a better understanding of the piRNA functions.

**Database URL:** http://www.regulatoryrna.org/database/piRNA/

## Introduction

piRNAs are a recently discovered class of small RNAs that bind to PIWI proteins. piRNAs are mainly expressed in the germline, although expression is also observed in somatic cells. In most species, the piRNAs range in size between 24 and 33 nt, whereas in *Caenorhabditis elegans,* the small RNAs corresponding to piRNAs are 21 nt in length and are commonly called 21U RNAs. piRNAs share a strong preference for a 5′-uridine residue. Genomic mapping have shown that piRNAs mostly originate from a limited number of clustered loci, each cluster being several kilobases in extension, and in which piRNAs may be encoded by one or both strands (1, 2). The amount of publicly available piRNA data is presently increasing rapidly.

piRNAs were first shown to function in post-transcriptional regulation of transposons. Reuter *et al*. (3) discovered that extensive complementarity between piRNAs and targeted transposon transcript was required for cleaving of targets in male germ cells by the protein MIWI, the mouse homologue of PIWI, and that the cleavage position was located 10 nt downstream of the 5′-end of the guide piRNA. Enrichment for L1- and IAP-derived piRNAs in mouse testes similarly showed a 10-nt distance between the 5′-ends of sense and antisense partners (4). Kiuchi *et al*. (5) found a 10-nt overlap between piRNAs derived from the Fem and Masc mRNAs in silkworm embryos, suggesting that piRNAs might participate in post-transcriptional silencing of coding genes by cleaving the corresponding mRNAs. In addition, piRNAs appear to induce mRNA deadenylation and decay in mouse elongating spermatids (6) and in the *Drosophila* embryo (7).

Epigenetic roles for piRNAs have also been discovered. In the fruit fly, PIWI binds to heterochromatin protein 1a (HP1a), which, upon methylation of histone H3K9, maintains the heterochromatin state of specific chromosomal regions (8, 9). It has also been reported that the PIWI protein can reactivate the euchromatin state of some chromosomal regions (10). Upon mutation of the PIWI proteins in mouse testes, the DNA methylation of retrotransposon genes is lost and the elements show increased expression (4, 11–13). Besides this, the levels of histone modification H3K9me3 on sequences flanking full-length L1-A copies were reduced in Miwi2 knockout spermatogonia (14). These results indicate that the piRNAs function in the establishment of DNA methylation and H3K9me3 marks on retrotransposons. Another report indicated that the Piwi/piRNA complex from the *Aplysia* central nervous system facilitates methylation of a conserved CpG island in the promoter of the breast cancer-related CERB2 gene (15).

The varied roles and rapidly increasing numbers of piRNAs underscore the need for a web analysis platform for piRNAs. In RNAcentral (16), the main database for RNA sequences, piRNABank (17) is the only piRNA database. Outside the RNAcentral, the piRNAQuest database (18) also focuses on piRNAs. As both piRNABank and piRNAQuest only contain limited amounts of piRNA data (Table 1) and annotations, and barely touches on the functions of the piRNAs, we have developed a new database named piRBase. piRBase has assembled a larger amount of piRNA data than the presently existing databases, and is the only database that includes epigenetic data and experimentally or computationally generated piRNA target data.

**Table 1.** Current numbers of unique piRNA sequences in piRBase and other piRNA databases

Species	piRNABank	piRNAQuest	piRBase
Human	32194	41749	32826
Mouse	72878	890078	51664769
Rat	62713	66758	63182
*D. melanogaster*	44417 ^a^	0	21027419
*C. elegans*	0	0	28219
Zebrafish	356550 ^a^	0	1330692
Chicken	0	0	508437
*X. tropicalis*	0	0	6142904
Silkworm	0	0	1174963
Platypus	147 ^a^	0	0

^a^The number of unique piRNAs may be less than shown.

**Figure 1.**
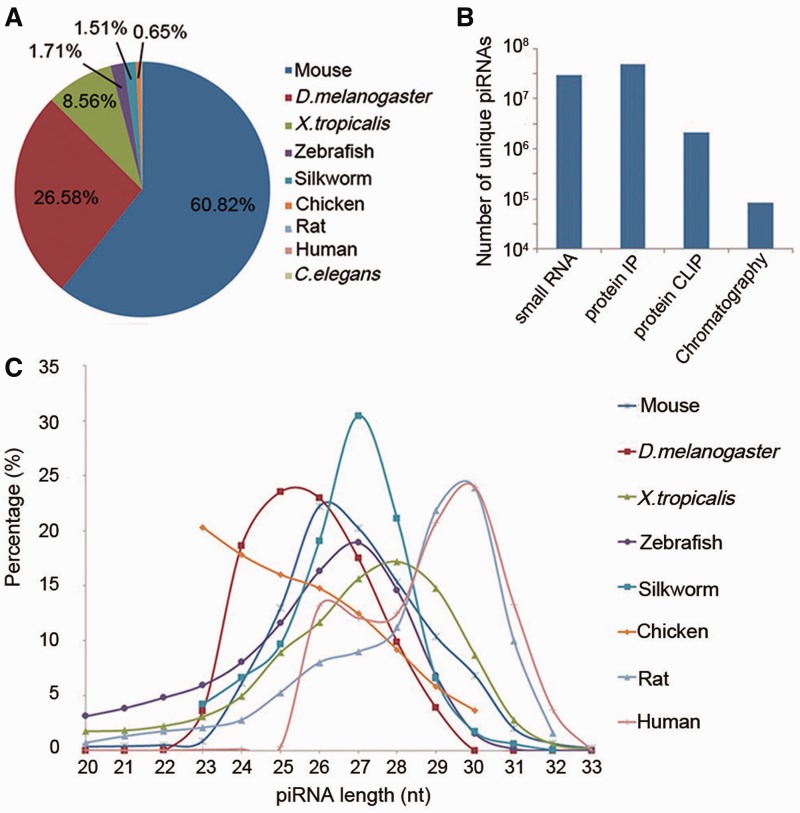
Overview of piRNA sequences in piRBase. (A) The percentage of unique piRNAs from each species in piRBase. (B) The amount of piRNA sequences obtained by different experimental methods. (C) Length distribution of unique piRNA sequences in piRBase. *Caenorhabditis elegans* piRNAs are not included as all reported sequences are 21 nt long.

Currently, piRBase contains 77 million piRNA sequences from nine organisms (Figure 1A), including data from worm (*C. elegans*), chicken, frog (*Xenopus tropicalis*) and silkworm (*Bombyx mori*) piRNAs which had previously not been collected by other piRNA databases. The amount of piRNA sequences derived from mouse, fruit fly and zebrafish is also much larger than in the other two databases (Table 1). More details on distinct piRNAs are provided, such as experimental method by which the piRNA was obtained, the tissues expressing the piRNAs, and annotations of the piRNA loci.

## Construction and content of the piRBase database

More than 77 million piRNA sequences and their corresponding annotations have been collected by piRBase. The data were collected from the literature and external databases. Processed piRNA sequences (txt or fasta files) have been preferred to raw sequencing data (sra or fastq files). We have put much effort into harvesting piRNA datasets from the literature and in verifying that these sequences were regarded as piRNAs by the authors of the respective papers. The piRNAs presently assembled in piRBase were mainly obtained by four experimental methods: (i) small RNA sequencing, (ii) immunoprecipitation of Piwi or Piwi-associated proteins, (iii) Piwi protein crosslinking-immunoprecipitation and (iv) chromatography. The amounts of piRNA sequences obtained by each method are displayed in Figure 1B. Figure 1C shows the length distribution of unique piRNA sequences in piRBase.

**Figure 2.**
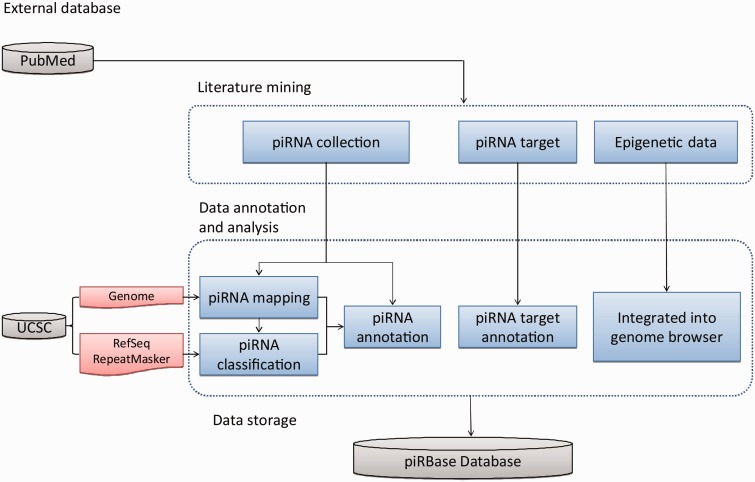
Database construction pipeline. The database was constructed in three major steps: manual literature mining, data annotation and analysis and data storage in a MySQL relational database with a Web interface.

After mapping the piRNAs to the genome, we took particular care to identify piRNAs that are derived from repeat elements and from coding genes, as these piRNAs might participate in the post-transcriptional regulation of the corresponding elements. In addition, piRBase also collected information on predicted and experimentally verified piRNA targets, DNA methylation data of tissues expressing piRNAs, and H3K9me3 data that may be related to piRNA function. The data collection and processing steps are illustrated in Figure 2 and in the Supplementary computational procedures.

### piRNA annotation

We have regarded the piRNA sequences from a separate RNA library as one dataset in piRBase. The piRNAs in piRBase are thus derived from more than 130 datasets (Supplementary Table S1). For every distinct piRNA sequence, we provide information including the piRBase piRNA name, NCBI and RNAdb piRNA aliases, NCBI piRNA accession number, organism of origin, sequence, sequence length, information on the datasets reporting the piRNA, PubMed id of the corresponding literature and the experimental method by which the piRNA was obtained. The piRBase piRNA name is unique for each piRNA record, and identical piRNA sequences from the same organism are combined as a single record.

In order to ascertain the origin of every piRNA sequence, we have mapped all piRNAs collected in piRBase to its corresponding genome using bowtie (19). No more than one mismatch was allowed, and only the best hits were reported (see Supplementary computational procedures for more detailed information).

### Data supporting functional analysis

#### Repeat/gene-derived piRNAs

According to the mapping result mentioned above, piRNAs mapping to RefSeq genes (20) or repeat elements annotated by RepeatMasker (21) are identified. These piRNAs are in piRBase referred to as gene- and repeat-derived piRNAs, respectively.

#### Post-transcriptional regulation data

Potential piRNA target genes with evidence of post-transcriptional regulation in mouse elongating spermatids (6) and in fruit fly embryos (7) were mined from the literature. For each piRNA–mRNA pair, we have recorded the piRNA, the region of the gene targeted by the piRNA and the piRNA functional mechanism. Experimentally verified piRNA–target relationships were noted. Thus far, this type of information only extends to mouse and fruit fly piRNA targets.

#### Epigenetic data

DNA methylation data for tissues expressing piRNAs were collected from the UCSC and GEO databases (22–25). The tissues include human brain, human testis, mouse brain, mouse testis, mouse spermatocytes, mouse spermatids, chicken testis, zebrafish testis and *Xenopus tropicalis* testis. Two forms of DNA methylation data have been collected: percentages of DNA methylation levels at the single-nucleotide scale, and non-methylated islands.

H3K9me3 ChIP-seq data for Miwi2 Het and Miwi2 KO mouse germ cells have been downloaded from the NCBI database to facilitate analysis of piRNA function in histone modification (14). The data supporting the functional analysis are listed in Table 2.

**Table 2.** List of data supporting the functional analysis in piRBase

Species	Repeat-/gene-derived piRNAs	Post-transcriptional piRNA target	DNA methylation data	H3K9me3 data
Human	Testis		Testis, brain	
Mouse	Testis, germ cell	Germ cell	Testis, germ cell, brain	Germ cell
Rat	Testis			
*D. melanogaster*	Testis, ovary	Embryo		
*C. elegans*	Whole worm			
Zebrafish	Ovary, testis		Testis	
Chicken	Embryo		Testis	
*X. tropicalis*	Egg, gastrula		Testis	
Silkworm				

### Data storage

In order to store the piRNA data and to facilitate piRNA function analysis, we constructed the piRBase Database and established a user-friendly Web interface. The piRBase is a MySQL relational database. The Web interface is built on PHP and JavaScript. For interactive data visualization, we have installed the UCSC Genome Browser (26). Alternatively, users can access the piRBase data from a download page and perform their own analyses.

## Web interface

### Browse and search piRNA annotations

#### Browsing piRNAs and datasets

**Figure 3.**
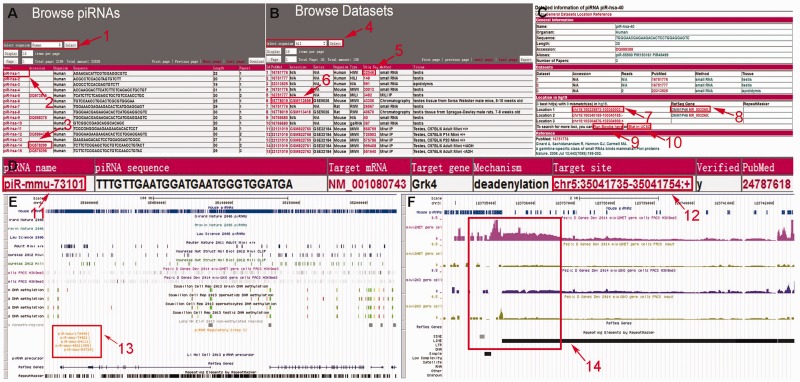
Screenshots of the browse and search pages. (A) The ‘Browse piRNAs’ page. (1) The drop down list box enables the users to browse piRNAs of a specific organism. (2 and 3) Clicking on the links delivers the detailed information page of the corresponding piRNA in piRBase and NCBI, respectively. (B) The ‘Browse Datasets’ page. The datasets can be filtered according to organism (4) and users can also browse piRNAs in a particular dataset (5). To learn more about the datasets, external links are provided (6). (C) In the piRNA ‘Detailed Information’ page, links to piRNA loci in the Genome Browser (7) and the annotations for these positions (8) are given. An online Bowtie tool (9) and a link to UCSC Blat (10) are also available for more alignment results. (D) In the piRNA target search result page, links to the piRNA ‘Detailed Information’ page of piRBase (11) and the target location in the Genome Browser (12) are available. (E and F) The locus information of the piRNAs, H3K9me3 marks, DNA methylation and piRNA target sites are shown in the Genome Browser. RefSeq genes and RepeatMasker annotations are also displayed. Screenshots of the Genome Browser show the piRNA target sites (13) and the H3K9me3 levels at a LINE1 locus (chr3:123735167-123741052 of mm9 genome) in Miwi2 HET and Miwi2 KO spermatogonia (14).

Users can browse the piRNAs by organism (Figure 3A) or browse the piRNAs of each individual dataset (Figure 3B). While browsing the piRNAs, detailed information on each piRNA is displayed by a click on the piRNA name. The detailed information page lists general information on the piRNA, the datasets containing the piRNA, its location in genome and the literature reporting it. The piRNA locus can be viewed in Genome Browser via the link in the detailed information page. The users can also view the piRNA description in NCBI by clicking on the accession number (Figure 3C).

#### Searching piRNAs

Using the web interface, the database can be searched by sequence, piRBase name, NCBI accession number and RNAdb name (27). Searching by sequence requires the complete piRNA sequence and allows up to two mismatches.

### Searching for data supporting functional analysis

#### Searching for repeat-/gene-derived piRNA

Search options for piRNAs derived from genes or repeats are also provided. The result pages are similar to the Browse result pages.

#### Searching for post-transcriptional regulation data

In order to support piRNA functional analysis, predicted and experimentally verified piRNA targets were collected. The web interface provides a piRNA target search module that users can use to search piRNA–target pairs by the name of functional piRNA, the target gene symbol or the RefSeq accession number. In the result page, a table is displayed that lists the basic information on functional piRNAs and target transcripts. In addition to the link to the detailed piRNA information, there is also a link to the Genome Browser showing the piRNA target sites in the genome (Figure 3D).

#### Searching for epigenetic data

Users can view DNA methylation levels and H3K9me3 levels at selected chromosome positions via an Epigenetics search module, and the DNA methylation levels of specific genes in the UCSC Genome Browser.

### The UCSC genome browser

Selected data are visualized in the Genome Browser in order to facilitate visual exploration (26), and can be accessed from each result page. This includes the piRNA locus, piRNA target sites and H3K9me3 and DNA methylation levels in specific tissues (28). In addition, some basic annotations from external databases, such as RepeatMasker annotations and RefSeq genes are included (Figure 3E and F). For example, to study the regulation of mRNA elimination by piRNAs, users can search piRBase by entering organism and piRNA name in the target mRNA search module. Detailed information on the piRNA–mRNA pair and a link to the Genome Browser will be displayed in the search result (Figure 3D), and the genomic positions corresponding to the piRNA-binding sites can be viewed in the Genome Browser by clicking on the link (Figure 3E). Pezic et al. (14) found that piRNAs target active LINE1s to establish repressive H3K9me3 marks in mouse spermatogonia. One of the reported LINE1s is located in chr3:123735167-123741052 of the mouse genome (mm9). Compared with Miwi2 KO spermatogonia, the H3K9me3 level of this region is higher in Miwi2 HET spermatogonia, and this is shown in the Genome Browser (Figure 3F).

### Downloading

The Download module provides two ways to download datasets. Users can either choose to download specific packages, or they can download piRNA data by submitting the piRBase piRNA name.

## Future directions

The number of piRNAs that are being reported is increasing rapidly. We will therefore update piRBase and integrate more information supporting piRNA functional analysis at intervals depending on the rate with which new data appear, expecting to issue new versions of the database about once every half year. In the future, we will also integrate piRNA datasets that provide only raw sequencing data. We will continue to develop the piRNA target prediction software, and special attention will be paid to the possibility of constructing piRNA-gene regulatory networks and elucidate piRNA action in distinct environments.

## Supplementary data

Supplementary data are available at *Database* Online.

Supplementary Data
