# Supplementary material for: piRBase: a web resource assisting piRNA functional study
Source: Database (Oxford). 2014 Nov 23;2014:bau110. doi: 10.1093/database/bau110 (PMC4243270; doi:10.1093/database/bau110)
Supplement: Supplementary Data [file supp_2014_bau110_index.html]

piRBase: a web resource assisting piRNA functional study — Supplementary Data 

# piRBase: a web resource assisting piRNA functional study

## Supplementary Data

files

**Files in this Data Supplement:**

- Supplementary Data - doc file
- Supplementary Data - xlsx file
