# Supplementary material for: piRBase: a web resource assisting piRNA functional study
Source: Database (Oxford). 2014 Nov 23;2014:bau110. doi: 10.1093/database/bau110 (PMC4243270; doi:10.1093/database/bau110)
Supplement: Supplementary Data [file supp_bau110_Supp_comp_proc2.doc]

# Supplementary computational procedures

piRNA dataset collection.

piRNA data were collected from the literature and external databases. Usually piRNA sequences from a distinct library are regarded as one dataset. There may thus be many datasets in one published paper. Identical piRNAs are merged and sequence reads are counted if available. Organism, tissue and experimental method are recorded for each dataset.

Importing piRNA datasets into piRBase.

For every unique piRNA sequence in the dataset, we first check whether this sequence already exists in the database for the given organism. If not, we add a new record for this piRNA including sequence, length and dataset information, and give it an ID/name. If the sequence already exists for the organism, only the dataset information for this record is modified.

Mapping piRNAs to genomes.

We did not require every piRNA in piRBase to map to a genome assembly by a specific criterion. For annotation, we mapped the piRNAs to the currently commonly used genome assemblies (mm9, hg19, rn4, dm3, ce10, galGal4, xenTro3 and danRer7) using bowtie v1.0 allowing at most 1 mismatch (bowtie -v 1 -a --best --strata). The number of best hits and mismatches for each piRNA were recorded. For piRNAs mapped to more than 10 loci in the genome, only 1 random position is retained. Aligned results are converted to BED6 format and uploaded into MySQL using the hgLoadBed tool provided by the UCSC genome browser. For human piRNAs, all repetitive mapping results were kept. For fruit fly piRNAs, another piRNA track showing piRNAs with no more than 200 loci were also generated.

For piRNA clusters reported according to a different genome version, we used liftOver tool provided by the UCSC genome browser to convert the coordinates. Unmapped records were removed.

piRNA classification

According to the above mapping results, we classified the piRNAs as gene or repeat related if one of the piRNA loci lies inside a RefSeq gene exon or a RepeatMasker annotated repeat element, respectively, in the corresponding genome assembly.

Epigenetic data

Tab delimited files containing DNA methylation levels based on bisulfite conversion at single nucleotide resolution and related ENCODE datasets were downloaded (table2). The tab delimited files were reformatted to BED12 and uploaded to MySQL. Since the processed H3K9me3 data available were mm10 based, we downloaded raw sequencing data and mapped them to the mm9 genome with bowtie v1.0 according to the authors’ criteria (bowtie -v 0 -a -m 10000), then reformatted the data to bigwig and uploaded them to the Genome Browser.
